# Allopurinol use predicts lower low-density lipoprotein cholesterol in patients with pre-dialysis chronic kidney disease—a prospective cohort study

**DOI:** 10.1093/ckj/sfae400

**Published:** 2024-12-09

**Authors:** Hulya Taskapan, Haley Ma, Berkay Taskapan, Paul Tam, Tabo Sikaneta

**Affiliations:** Research Department, Kidney Life Sciences Institute, Toronto, Canada; Research Department, Kidney Life Sciences Institute, Toronto, Canada; Research Department, Kidney Life Sciences Institute, Toronto, Canada; Research Department, Kidney Life Sciences Institute, Toronto, Canada; Department of Nephrology, The Scarborough Health Network, Toronto, Canada; Research Department, Kidney Life Sciences Institute, Toronto, Canada; Department of Nephrology, The Scarborough Health Network, Toronto, Canada; Department of Medicine, University of Toronto, Canada

**Keywords:** allopurinol, cardiovascular, CKD, dyslipidemia, uric acid

## Abstract

**Background:**

Hyperuricemia influences lipid metabolism, yet relationships between urate-lowering therapy with allopurinol, serum urate, and lipid levels in patients with chronic kidney disease remain underexplored.

**Methods:**

This was a post-hoc analysis of 1970 participants of the CAN AIM to PREVENT who had pre-dialysis chronic kidney disease and were not receiving lipid-lowering therapy or febuxostat. Joint generalized structural equation modeling was used to investigate associations between allopurinol use (yes or no), serum urate [as a continuous or categorical variable (target if <6 mg/dl or high if ≥6 mg/dl)], and lipid levels [total cholesterol, low-density lipoprotein-cholesterol (LDL-C), high-density lipoprotein cholesterol, and triglycerides) assessed every 6 months for up to 3 years, along with time-to-event outcomes (death or initiation of renal replacement therapy), adjusting for demographic and clinical factors. Mediation analysis was used to determine allopurinol's direct and indirect effects (via urate) on lipid levels.

**Results:**

Allopurinol use independently predicted lower total cholesterol (–7.94%, 95% CI: −12.13% to –3.54%, *p *< 0.001) and LDL-C [–13.84% (–21.14 to –5.87), *p *= 0.001]. Serum urate independently predicted a small increase in LDL-C [0.02% per mg/dl (0.009 to 0.03), *p *< 0.001]. Patients on allopurinol with target urate had lower LDL-C compared to those not on allopurinol with target urate [–4.46% (–8.25 to –0.50), *p *= 0.027] and those on allopurinol with high urate [–10.15% (–13.16 to –7.04), *p *< 0.001]. Mediation analysis showed that serum urate indirectly mediated only 24% of the effect of allopurinol on LDL-C.

**Conclusion:**

Allopurinol use predicted lower total and especially LDL cholesterol independently of serum urate in this cohort of patients with pre-dialysis chronic kidney disease. Future studies could investigate underlying mechanisms, evaluate clinical implications, and confirm these findings in this and other populations.

## Graphical Abstract

**Graphical Abstract:**
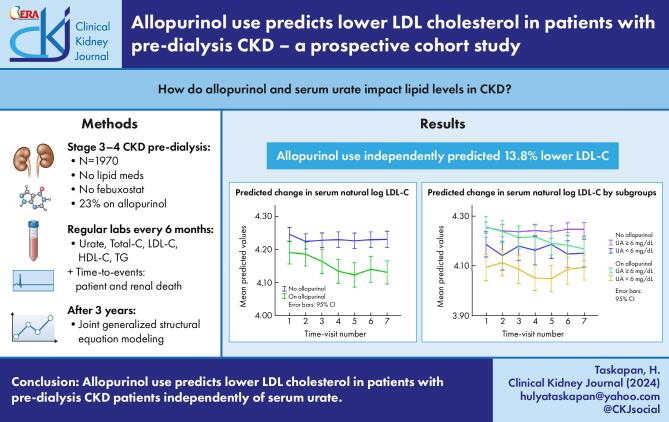

KEY LEARNING POINTS **What was known:**

- Associations have been reported between urate concentrations and/or use of xanthine oxidase inhibitors with cholesterol levels in animal and human studies.
- The latter were limited by examination of patients who were already on traditional lipid-lowering therapy, and exclusion of patients with advanced CKD.

**This study adds:**

- Confirmation that allopurinol use predicted a 14% lowering of LDL cholesterol in patients with stage 3 and 4 CKD who were not receiving traditional lipid-lowering therapy. This effect was maximal in patients whose urate levels were lowered to below 6 mg/dL, suggesting a possible correlation with effective xanthine oxidase inhibition.
- Confirmation of a much weaker inverse association between urate and cholesterol levels.

**Potential impact:**

- The study findings suggest that allopurinol use independently predicts lower LDL cholesterol in patients with pre-dialysis CKD, particularly when urate concentrations are lowered to below 6 mg/dL.
- Future studies could determine mechanisms, clinical implications, and generalizability to other populations.

## INTRODUCTION

As two-thirds of the daily urate produced in humans is excreted by the kidneys, hyperuricemia and gout occur more frequently in patients with chronic kidney disease (CKD). These patients are consequently prescribed urate-lowering therapy more often than people without CKD [1]. Allopurinol, the most widely used urate-lowering agent, inhibits xanthine oxidase and reduces the conversion of hypoxanthine to xanthine and xanthine to urate. Emerging clinical and epidemiological evidence suggests that hyperuricemia may also contribute to the heightened prevalence of dyslipidemia observed in this population [2]. Furthermore, urate-lowering therapy has been shown to lower lipid levels in animal studies [3, 4, 5], and low-density lipoprotein cholesterol (LDL-C) in patients with mild CKD [6, 7, 8]. However associations between allopurinol use, serum urate, and lipid levels remain understudied as these studies were limited by examining urate-lowering therapy effects on lipids in the presence of lipid-lowering therapy; failure to stratify by whether target urate levels were achieved; heterogeneous patient populations; brief follow-up durations; suboptimal study designs; mixed results; and exclusion of patients with CKD [6, 7, 8].

In this study, we examined associations between allopurinol use, serum urate, and serum lipids [total cholesterol, LDL-C, high-density lipoprotein cholesterol (HDL-C), and triglycerides] in a large prospective cohort of multiethnic patients with pre-dialysis CKD living in Toronto Canada.

## MATERIALS AND METHODS

### Study design and ethics considerations

The CAN AIM to PREVENT Trial was an investigator-initiated, prospective, open observational cohort study conducted at three pre-dialysis clinics in Toronto between 2010 and 2015 [9]. The primary aim was to examine rates of progression to dialysis in relation to inflammatory markers. All subjects provided signed consent for the original study and as a post-hoc analysis, no additional consent was obtained for this study. The original study was registered on ClinicalTrials.gov (NCT01974713).

### Study population and data collection

Participants enrolled in the CAN AIM to PREVENT had estimated glomerular filtration rate (eGFR) less than 60 ml/min, had never been on dialysis or received a renal transplant, were over the age of 18, did not have a terminal illness, and were able to provide informed consent. They were recruited by their regular nephrologists and provided signed informed consent before inclusion. Comprehensive demographic data and clinical histories were collected at the outset. Additionally, details on medication use, vital signs, and laboratory values, including serum concentrations of urate and lipid profiles, were systematically recorded at baseline and subsequently every 6 months for up to 3 years. Patients analyzed in the current study were restricted to those with eGFR 15–60 ml/min/1.73 m^2^ at study enrolment, and who were not prescribed febuxostat or lipid-lowering agents at any point in the study.

### Exposures

Allopurinol use (yes or no) and serum urate (target if <6 mg/dl or high if ≥6 mg/dl).

### Outcomes

Serum lipids (total cholesterol, LDL-C and HDL-C, and triglycerides).

### Statistical analysis

All data analyses were performed using Stata software (StataCorp. 2023, Stata Statistical Software: Release 17, College Station, TX: StataCorp LP). Descriptive statistics, including means, medians, and frequencies, were employed to summarize the data. Kruskal–Wallis tests and analysis of variance were used to compare baseline characteristics and laboratory values across groups. Chi-square tests were used for comparing categorical variables, with a significance level set at *p* < 0.05. Non-normally distributed variables such as each examined lipid, body mass index, C-reactive protein, and urine protein/creatinine ratio were naturally log-transformed to ensure symmetric distributions.

To evaluate relationships between allopurinol use and urate levels with both lipid levels and time-to-event data, joint generalized structural equation modeling (GSEM) was employed. This allowed for the simultaneous assessment of continuous and time-to-event outcomes within a single framework, incorporating shared random effects across outcomes. Lipid levels were modeled using a Gaussian family with an identity link, whereas time-to-event data utilized a Weibull family and log link, adjusted for single-censoring events. Patient and renal death were the event outcomes censured in the time-to-event analyses. Covariates included allopurinol and urate categories, baseline diabetes status, age, sex, eGFR, serum albumin, body mass index (BMI), urinary albumin-to-creatinine ratio, and C-reactive protein, along with follow-up visit number and its square. A latent variable captured unmeasured heterogeneity across subjects, and robust standard errors addressed within-subject correlation. Differences in lipid levels were then presented as percentages calculated using exponentials of model-predicted beta-coefficients [(e^β^–1) × 100]. Differences in censured events were expressed as hazard ratios calculated by exponentiating beta coefficients (e^β^).

To determine allopurinol's direct and indirect effects (via serum urate) on lipid levels, mediation analysis was performed. The Baron and Kenny approach as provided in the structural equation modeling package by Stata was used. Effects are represented as percentages.

To assess the potential impact of a baseline history of gout on examined lipid and time-to event outcomes, joint GSEM was repeated among allopurinol users after stratification by history of gout at study entry.

## RESULTS

### Participant selection and baseline characteristics

A total of 2254 patients enrolled in the CAN AIM to PREVENT. Two hundred eighty-four were prescribed lipid-lowering agents or febuxostat at some point during the study and were excluded from this analysis. Of the remaining 1970 patients, 69.5% were male, 48.7% had diabetes mellitus, the mean age was 69.3 years (SD 12.1), the mean eGFR was 38.6 ml/min/1.73m² ± 11.2, and the mean serum urate was 7.5 mg/dl (SD 1.9). Allopurinol was prescribed to 445 (22.6%) participants at baseline, with unadjusted differences in baseline characteristics stratified by allopurinol use as shown (Table 1). The unadjusted differences in baseline characteristics after stratification by allopurinol use and urate category are presented in Table 2.

**Table 1:** **Participant baseline characteristics by allopurinol use**.

**Variable**	**Total (*n* = 1970)**	**Not on allopurinol (*n* = 1525)**	**On allopurinol (*n* = 445)**	***p*-Value**
Age (years)	69.3 ± 12.1	69.2 ± 12.4	70.6 ± 11.0	0.1135
% Male	69.5	62.5	78.2	**<0.001**
% with gout	24.1	9.3	74.6	**<0.001**
% with diabetes mellitus	48.7	49.9	43.8	**0.024**
BMI	28.1 ± 5.6	27.8 ± 5.5	28.8 ± 5.6	**0.0012**
eFR (/min/1.73 m^2^)	38.6 ± 11.2	39.0 ± 11.3	37.4 ± 10.8	**0.0095**
Albumin (g/dl)	4.3 ± 0.3	4.3 ± 0.3	4.3 ± 0.4	0.9179
Urate (mg/dl)	7.5 ± 1.9	7.79 ± 1.86	6.6 ± 1.7	**<0.001**
Total cholesterol (mg/dl)	160.5 ± 43.3	161.3 ± 44.2	157.7 ± 40.1	0.2004
LDL (mg/dl)	76.3 ± 34.3	77.1 ± 35.2	73.6 ± 31.1	0.1866
HDL (mg/dl)	50.6 ± 16.9	51.2 ± 16.0	48.73 ± 19.0	**0.0002**
Triglycerides (mg/dl)	171.7 ± 113.9	168.2 ± 113.1	183.8 ± 116.0	**0.0017**
C-reactive protein (mg/l)	3.7 ± 7.1	3.6 ± 7.4	3.9 ± 7.9	**0.0083**
Urine albumin/creatinine (mg/mmol)	48.8 ± 100.0	56.4 ± 42.0	45.6 ± 100.2	**0.0003**

**Table 2:** Participant baseline characteristics by allopurinol* and urate categories**.

**Variable**	**NA, TU (*n* = 238)**	**NA, HU (*n* = 1287)**	**A, HU (*n* = 264)**	**A, TU (*n* = 181)**	***p*-Value**
Age (years)	70.6 ± 11.3	68.9 ± 12.5	68.2 ± 11.5	73.9 ± 9.4	**0.0001**
% Male	52.6	64.3	79.3	76.3	**<0.001**
% with gout	4.62	10.23	73.11	77.22	**<0.001**
% with diabetes mellitus	50.6	49.8	43.4	44.9	0.169
BMI	26.4 ± 4.7	28.1 ± 5.6	29.3 ± 5.7	28.1 ± 5.4	**0.001** a,b,c,d,f
eGFR (/min/1.73 m^2^)	43.7 ± 10.9	38.0 ± 11.2	38.34 ± 10.89	36.05 ± 10.63	**<0.001** a,b,c
Albumin (g/dl)	4.3 ± 0.3	4.3 ± 0.3	4.3 ± 0.4	4.3 ± 0.3	0.9823
Urate (mg/dl)	5.1 ± 0.9	8.3 ± 1.5	7.7 ± 1.3	5.1 ± 0.7	**< 0.001**
Total cholesterol (mg/dl)	159.0 ± 42.7	161.78 ± 44.5	166.5 ± 41.8	145.4 ± 34.5	**0.001** a, c, f
LDL (mg/dl)	72.8 ± 32.5	77.9 ± 35.7	79.69 ± 33.11	65.59 ± 26.44	**0.001** a,c,e,f
HDL (mg/dl)	55.8 ± 17.5	50.3 ± 15.6	48.4 ± 16.0	48.8 ± 22.4	**0.001** a,b,c,d,e
Triglycerides (mg/dl)	146.8 ± 95.7	172.3 ± 115.7	198.7 ± 126.3	164.8 ± 97.8	**0.001** a,b,c,d,f
C reactive protein (mg/l)	3.0 ± 6.9	3.7 ± 7.4	3.6 ± 4.4	4.3 ± 7.9	0.0406 a,b,c,d,f
Urine albumin/creatinine (mg/mmol)	35.9 ± 93.8	47.4 ± 101.4	64.70 ± 98.43	43.3 ± 70.3	**< 0.001** a,b,c,d,e

*NA (no allopurinol), A (on allopurinol).

**TU (target urate, <6 mg/dl), and HU (high urate, ≥ 6 mg/dl).

a, TU, NA versus HU, NA; b, TU, NA versus HU, A; c, TU, NA versus TU, A; d, HU, NA versus HU, A; e, HU, NA versus TU, A; f, HU, A versus TU, A.

Values in bold indicate statistical significance with a *p*-Value < 0.05.

### Patient and renal survival during the study

Two hundred eight (10.6%) patients reached the time-to-event censuring endpoint of death (130 patients) or initiation of renal replacement therapy (78 patients). This occurred in 10.62% of patients not on allopurinol, and 10.34% of those on allopurinol (*p* = 0.932).

### Changes in allopurinol use and urate categories during the study

An additional 35 patients started taking allopurinol during the study. This was not associated with the use of thiazide or loop diuretics. The numbers of unique patients in each urate category during the follow-up period were 262 with target urate and not on allopurinol; 1735 with high urate and not on allopurinol; 287 with high urate and on allopurinol; and 193 with target urate and on allopurinol. Patients not prescribed allopurinol had 1341 study visits with target and 11 522 visits with high urate levels, respectively, whereas patients prescribed allopurinol had 1283 visits with target and 1791 visits with high urate levels, respectively. Urate levels were at target in 41.7% of study visits for patients on allopurinol and 12.0% for those not on allopurinol (*p *< 0.001 for difference).

### Lipids in relation to allopurinol use and serum urate (Table 3, Figs. 1–4)

Allopurinol use was associated with lower total cholesterol [7.94% (95% CI –12.13 to –3.54), *p *= 0.001] and serum urate (as a continuous variable) was associated with a very small elevation in total cholesterol [0.007% per mg/dl (0.0001 to 0.01), *p *= 0.045]. The interaction between allopurinol use and serum urate was also associated with total cholesterol [0.02% per mg/dl (0.008 to 0.03), *p *= 0.001]. Allopurinol use was associated with lower LDL-C [−13.84% (−21.14 to −5.87), *p *= 0.001] and serum urate with higher LDL-C [0.02% per mg/dl (0.009 to 0.03), *p *< 0.001]. The interaction between allopurinol use and serum urate also influenced LDL-C [0.03% per mg/dl (0.01 to 0.05), *p *= 0.004]. Both allopurinol use and serum urate were associated with lower HDL-C [–1.62% (–2.99 to –0.23), *p *= 0.022; and 0.008% per mg/dl (–0.01 to –0.003), *p *= 0.004, respectively]. Allopurinol use and serum urate were also both associated with higher triglyceride levels [6.23% (3.02 to 9.53), *p *< 0.001; and 0.2% per mg/dl (0.008 to 0.03), *p *= 0.001, respectively].

**Figure 1:**
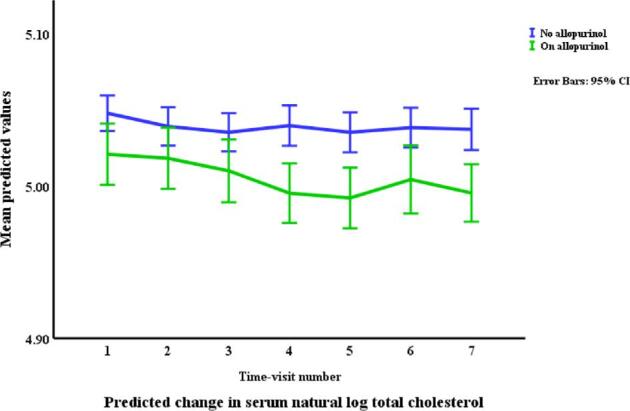
Changes* in total cholesterol by allopurinol use. *Based on a generalized structural equation model predicting changes in serum total cholesterol adjusted for age, gender, BMI, visit number (as a proxy for time), eGFR, history of diabetes mellitus, use of allopurinol, serum albumin, C-reactive protein, urate, the interaction between urate and use of allopurinol, and urine albumin/creatinine ratio.

**Figure 2:**
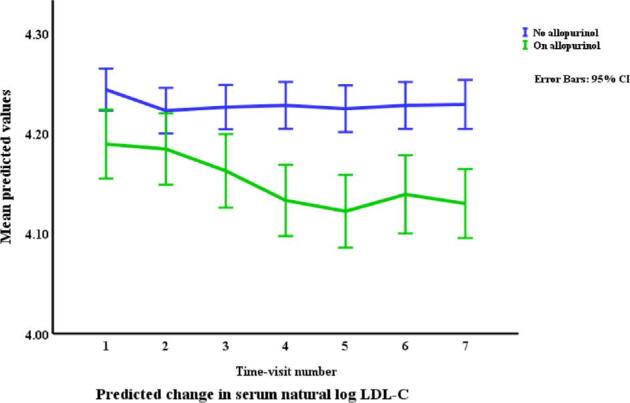
Changes* in LDL-C by allopurinol use. *Based on a generalized structural equation model predicting changes in LDL-C adjusted for age, gender, BMI, visit number (as a proxy for time), eGFR, history of diabetes mellitus, use of allopurinol, serum albumin, C-reactive protein, urate, the interaction between urate and use of allopurinol, and urine albumin/creatinine ratio.

**Figure 3:**
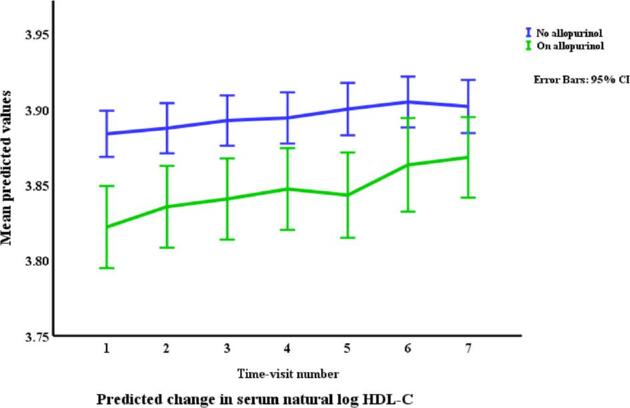
Changes* in HDL-C by allopurinol use. *Based on a generalized structural equation model predicting changes in serum HDL adjusted for age, gender, BMI, visit number (as a proxy for time), eGFR, history of diabetes mellitus, use of allopurinol, serum albumin, C-reactive protein, urate, the interaction between urate and use of allopurinol, and urine albumin/creatinine ratio.

**Figure 4:**
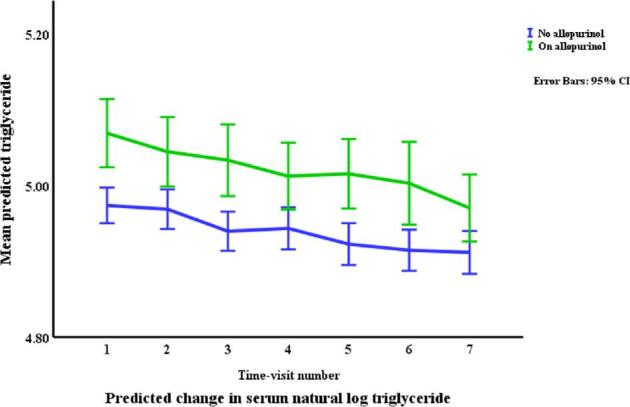
Changes* in triglycerides by allopurinol use. *Based on a generalized structural equation model predicting changes in serum triglyceride adjusted for age, gender, BMI, visit number (as a proxy for time), eGFR, history of diabetes mellitus, use of allopurinol, serum albumin, C-reactive protein, urate, the interaction between urate and use of allopurinol, and urine albumin/creatinine ratio.

**Figure 5:**
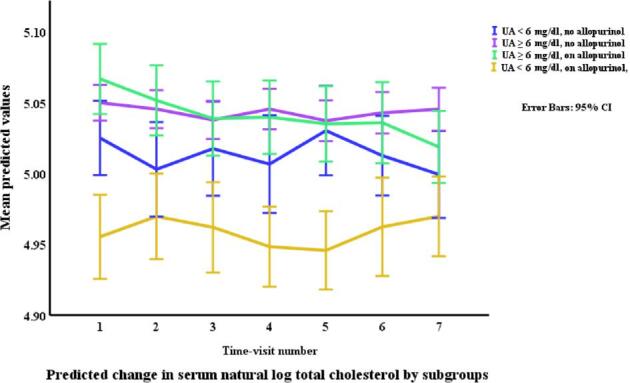
Changes* in total cholesterol by allopurinol use and urate category. *Based on a generalized structural equation model predicting changes in serum total cholesterol adjusted for age, gender, BMI, visit number (as a proxy for time), eGFR, history of diabetes mellitus, serum albumin, C-reactive protein, and urine albumin/creatinine ratio.

**Figure 6:**
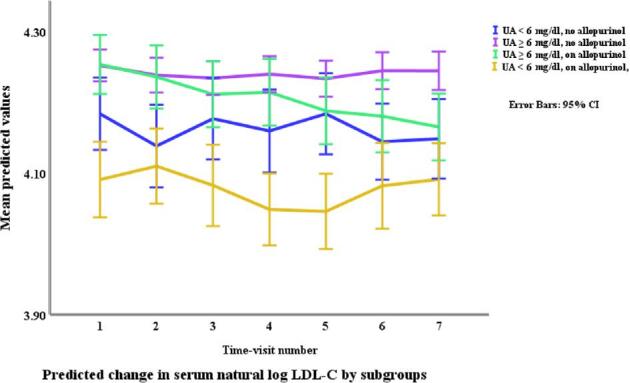
Changes* in LDL-C by allopurinol use and urate category. *Based on a generalized structural equation model predicting changes in serum LDL adjusted for age, gender, BMI, visit number (as a proxy for time), eGFR, history of diabetes mellitus, serum albumin, C-reactive protein, and urine albumin/creatinine ratio.

**Figure 7:**
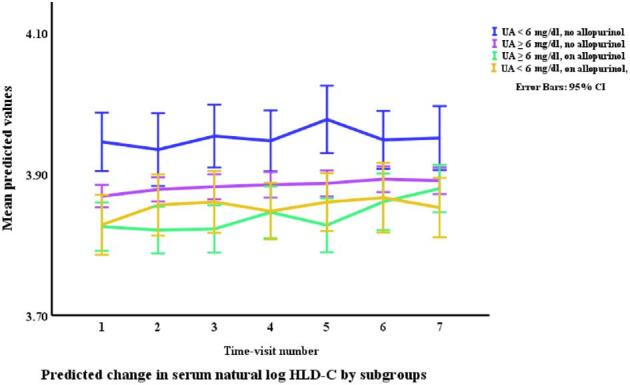
Changes* in HDL-C by allopurinol use and urate category. *Based on a generalized structural equation model predicting changes in serum HDL adjusted for age, gender, BMI, visit number (as a proxy for time), eGFR, history of diabetes mellitus, serum albumin, C-reactive protein, and urine albumin/creatinine ratio.

**Figure 8:**
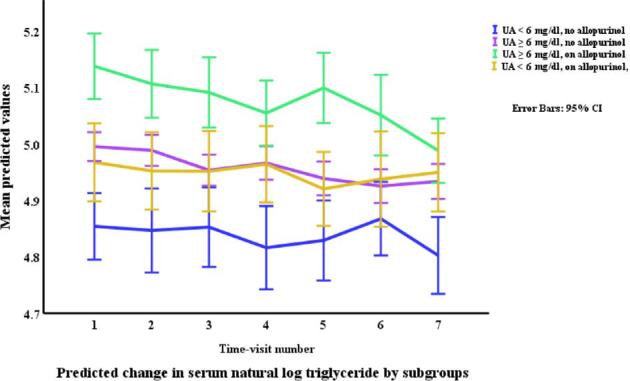
Changes* in triglycerides by allopurinol use and urate category. *Based on a generalized structural equation model predicting changes in serum triglyceride adjusted for age, gender, BMI, visit number (as a proxy for time), eGFR, history of diabetes mellitus, serum albumin, C-reactive protein, and urine albumin/creatinine ratio.

**Table 3:** Allopurinol use and serum urate (as a continuous variable) as predictors of lipid levels and censured events (patient and renal death) in a GSEM.

**Independent** **variable**	**Total cholesterol** **(95% CI)** ***p*-value**	**LDL-C** **(95% CI)** ***p*-value**	**HDL-C** **(95% CI)** ***p*-value**	**Triglycerides** **(95% CI)** ***p*-value**
% Change in lipid levels				
Allopurinol use (yes)	–7.94 (–12.13 to –3.54) 0.001	−13.84 (−21.14 to −5.87) 0.001	–1.62 (–2.99 to –0.23) 0.022	6.23 (3.02 to 9.53) <0.001
Serum urate (per mg/dl)	0.007 (0.0001 to 0.01) 0.045	0.02 (0.009 to 0.03) <0.001	–0.008 (–0.01 to –0.003) 0.004	0.02 (0.008 to 0.03) 0.001
Allopurinol urate interaction	0.02 (0.008 to 0.03) 0.001	0.03 (0.01 to 0.05) 0.004	–	–
Hazard ratios for censured events				
Allopurinol use (yes)	3.02 (0.64 to 14.36) 0.165	3.39 (0.75 to 15.30) 0.112	1.38 (0.89 to –2.15) 0.150	1.39 (0.89 to 2.17) 0.151
Serum urate (per mg/dl)	1.00 (0.99 to 1.00) 0.196	1.00 (0.99 to 1.00) 0.130	1.00 (0.99 to –1.00) 0.215	1.00 (0.99 to 1.00) 0.207
Allopurinol urate interaction	1.00 (1.00 to 1.00) 0.289	0.99 (0.99 to 1.00) 0.200	–	–

*Adjusted for age, gender, BMI, visit number (as a proxy for time), eGFR, diabetes history, serum albumin, C reactive protein, and microalbumin/creatinine ratio, with an unstructured covariance and robust standard errors.

**Differences in lipid levels expressed as percentages and calculated using exponentials of model-predicted beta-coefficients: (e^β^–1) × 100. Differences in censured events expressed as hazard ratios and calculated by exponentiating beta coefficients (e^β^).

### Lipids in relation to allopurinol use stratified by urate category (Table 4, Figs. 5–8)

Patients not on allopurinol with high urate had significantly higher total cholesterol compared to those not on allopurinol with target urate [2.24% (0.68 to 3.46), *p *= 0.005]. There was no difference in total cholesterol in patients on allopurinol with target urate compared to those not on allopurinol with target urate [–2.06% (–4.41 to 0.04), *p *= 0.055]. Patients on allopurinol with target urate had significantly lower total cholesterol compared to those not on allopurinol with high urate [–4.20% (–5.86 to –2.52), *p *< 0.0001] and compared to those on allopurinol with high urate [–4.51% (–6.02 to –2.97), *p *< 0.0001]. Patients on allopurinol with high urate had significantly higher total cholesterol [2.56% (0.34 to 4.83), *p *= 0.023] compared those not on allopurinol with target urate. Patients not receiving allopurinol with high urate had significantly higher LDL-C compared to those not on allopurinol with target urate [6.34% (3.38 to 9.38), *p *< 0.0001]. Conversely, patients on allopurinol with target urate exhibited a significant reduction in LDL-C compared to all other groups [–4.46% (–8.25 to –0.50), *p *= 0.027] compared to those not on allopurinol with target urate [–10.15% (–13.16 to –7.04), *p *< 0.0001] compared to those not on allopurinol with high urate; and [–8.0% (–10.86 to –5.06), *p *< 0.0001] compared to those on allopurinol with high urate. Patients on allopurinol with target urate had significant reductions in HDL-C compared to those not on allopurinol with target urate [–2.33% (–4.16 to –0.47), *p *= 0.014]. No significant differences in HDL-C were observed in the other comparison groups. Patients on allopurinol with high urate had higher triglyceride levels compared to those not on allopurinol with target urate [8.30% (4.09 to 12.69), *p *< 0.0001]. Triglyceride levels were higher in patients on allopurinol with target urate compared to those not on allopurinol with target urate [5.13% (0.68 to 9.78), *p *= 0.023]. Patients on allopurinol with high urate had higher triglycerides than patients not on allopurinol with high urate [5.42% (2.12 to 8.82), *p *= 0.001]. No significant differences were observed in the other comparison groups.

### Patient and renal deaths (Tables 3 and 4)

Allopurinol use and serum urate levels did not predict the combined censured endpoint of patient and renal death in any of the joint GSEMs tested.

**Table 4:** **Allopurinol use and urate categories as predictors of lipid levels and censured events (patient and renal death) in a GSEM**.

**Comparison** groups***	**Total cholesterol (95% CI)** ***p*-value**	**LDL-C** **(95% CI)** ***p*-value**	**HDL-C** **(95% CI)** ***p*-value**	**Triglycerides** **(95% CI)** ***p*-value**
% Change in lipid levels				
NA, HU versus NA, TU	2.24 (0.68 to 3.46) **0.005**	6.34 (3.38 to 9.38) **<0.001**	−1.20 (–2.52 to 0.14) 0.080	2.47 (–0.06 to 5.62) 0.055
A, HU versus NA, TU	2.56 (0.34 to 4.83) **0.023**	3.86 (–0.036 to 7.90) 0.052	–1.56 (–3.35 to 0.26) 0.093	8.30 (4.09 to 12.69) **<0.001**
A, TU versus NA, TU	–2.06 (–4.41 to 0.04) **0.055**	–4.46 (–8.25 to –0.50) **0.027**	–2.33 (–4.16 to –0.47) **0.014**	5.13 (0.68 to 9.78) **0.023**
A, TU versus NA, HU	–4.20 (–5.86 to –2.52) **<0.001**	–10.15 (–13.16 to –7.04) **<0.001**	–1.15 (–2.76 to –0.50) 0.172	2.33 (–1.42 to 6.21) 0.226
A, TU versus A, HU	–4.51 (–6.02 to –2.97) **<0.001**	–8.0 (–10.86 to –5.06) **<0.001**	–0.78 (–2.25 to 0.69) 0.296	–2.93 (–5.99 to 0.24) 0.069
A, HU versus NA, HU	0.32 (–1.39 to 2.05) 0.719	–2.33 (–5.25 to 37.37) 0.383	–0.37 (–1.83 to 1.11) 0.623	5.42 (2.12 to 8.82) **0.001**
Hazard ratios for censured events				
NA, HU v ersus NA, TU	0.80 (0.45 to 1.41) 0.436	0.79 (0.44 to 1.40) 0.419	0.98 (0.44 to 1.42) 0.441	0.80 (0.45 to 1.44) 0.467
A, HU versus NA, TU	0.63 (0.30 to 1.32) 0.218	0.61 (0.29 to 1.28) 0.194	0.61 (0.29 to 1.28) 0.192	0.62 (0.29 to 1.31) 0.212
A, TU versus NA, TU	1.06 (0.53 to 2.10) 0.867	1.05 (0.53 to 2.08) 0.888	1.08 (0.54 to 2.15) 0.836	1.07 (0.54 to 2.15) 0.841
A, TU versus NA, HU	1.33 (0.81 to 2.19)	1.33 (0.81 to 2.19) 0.261	1.35 (0.82 to 2.23) 0.237	1.02 (0.99 to 0.94) 0.226
A, TU versus A, HU	1.69 (0.85 to 3.37) 0.137	1.29 (0.73 to 2.29)	1.0 (0.98 to 1.0) 0.296	1.73 (0.86 to 3.45) 0.122
A, HU versus NA, HU	0.79 (0.44 to 1.40) 0.413	0.78 (0.44 to 1.37) 0.383	0.76 (0.43 to 1.36) 0.358	0.77 (0.44 to 1.37) 0.375

*Adjusted for age, gender, BMI, visit number (as a proxy for time), eGFR, diabetes history, serum albumin, C-reactive protein, and microalbumin/creatinine ratio, with an unstructured covariance and robust standard errors.

**Differences in lipid levels expressed as percentages and calculated using exponentials of model-predicted beta-coefficients: (e^β^–1) × 100. Differences in censured events expressed as hazard ratios and calculated by exponentiating beta coefficients (e^β^).

*** NA (no allopurinol), A (on allopurinol), TU (target urate, <6 mg/dl) and HU (high urate, ≥ 6 mg/dl).

### Mediation analysis (Table 5)

Serum urate mediated 30.1% of the effect of allopurinol on total cholesterol [indirect effect: –0.01 (–0.013 to –0.006), *p *< 0.001] and 24.3% of allopurinol's effect on LDL-C [indirect effect: –0.02 (–0.026 to –0.014), *p *< 0.001]. Serum urate mediated 51.1% of the effect of allopurinol on HDL-C [indirect effect: 0.025 (0.020 to 0.029), *p *< 0.001] and 54% of the effect on triglycerides [indirect effect: –0.044 (–0.052 to –0.037), *p *< 0.001].

**Table 5:** Mediation analysis summary for the effect of urate on lipid parameters.

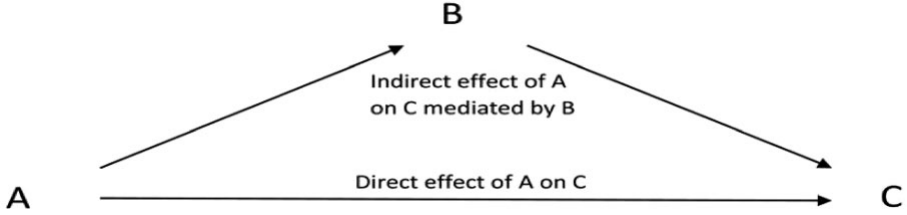						
**A**	**B**	**C**	**Indirect effect (95% CI)**	**Direct effect (95% CI)**	***p*-Value**	**Mediation by serum urate (%)**
Allopurinol	Serum urate	Total cholesterol	–0.01 (–0.013, –0.006)	–0.02 (–0.03, –0.01)	0.000	30.1
Allopurinol	Serum urate	LDL-C	–0.02 (–0.026, –0.014)	–0.062 (–0.08, –0.04)	0.000	24.3
Allopurinol	Serum urate	HDL-C	0.025 (0.020, 0.029)	–0.073 (–0.09, –0.06)	0.000	51.1
Allopurinol	Serum urate	Triglycerides	–0.044 (–0.052, –0.037)	0.125 (0.101, 0.149)	<0.001	54

### Subgroup analysis by history of gout (Supplementary Tables 1 and 2)

A history of gout at study baseline was not associated with any of the examined lipid levels or time-to-event outcomes among patients treated with allopurinol.

## DISCUSSION

We examined the associations between allopurinol use, serum urate, and lipid levels in this multicenter prospective cohort of 1970 multiethnic individuals with pre-dialysis CKD who were not prescribed lipid-lowering therapy or febuxostat. We found that participants prescribed allopurinol had total cholesterol and LDL-C levels that were 7.94% and 13.84% lower, respectively, than those not prescribed allopurinol, and that although this was independent of serum urate, lower urate also independently predicted lower total cholesterol and LDL-C. Patients who were prescribed allopurinol and had serum urates below 6 mg/dl had the lowest LDL-C. Mediation analysis confirmed a strong direct and, via serum urate, a three-fold weaker indirect effect of allopurinol on serum LDL-C. The results suggest that allopurinol, especially when serum urate is lowered to below 6 mg/dl, can reduce serum LDL-C in patients with pre-dialysis CKD.

The synergism between allopurinol use and serum urate <6 mg/dl suggests that effective xanthine oxidase inhibition may be central to allopurinol–LDL-C reduction. Xanthine oxidase activity is reported to directly and indirectly alter gastrointestinal lipid absorption, hepatic cholesterol synthesis, and clearance of lipoproteins. For example, murine knock-in studies have shown xanthine oxidase overexpression increases gastrointestinal absorption and transport of excess dietary fat, and that this is reduced with allopurinol [10]. Additionally, xanthine oxidase can generate oxidative stress and promote inflammation, both of which indirectly affect lipid metabolism, including cholesterol synthesis and regulation [11, 12]. However effective xanthine oxidase inhibition appears to be relatively uncommon. In the current study, urate levels less than 6 mg/dl in allopurinol recipients were seen at only 41.7% of study visits. Even lower rates of 19–32% are reported among allopurinol recipients in the general population [13–15, 16]. Thus, the majority of patients prescribed allopurinol fail to attain this urate target as set by the European League Against Rheumatism [17] and the American College of Rheumatology (ACR) guidelines [18]. This might be explained by dietary non-compliance and/or by allopurinol under-dosing (related to patient non-compliance, and the lack of target urate levels [19] or hesitancy of practitioners to titrate doses to urate targets in patients with CKD) [20]. Genetically mediated allopurinol resistance might also contribute as single nucleotide polymorphisms have been shown to alter allopurinol absorption, metabolism, excretion, and drug–drug interactions [21].

The clinical implications of allopurinol–LDL-C reduction especially with regard to cardiovascular outcomes are unknown. People with CKD suffer disproportionately high rates of cardiovascular mortality and morbidity, and the dyslipidemia of CKD is increasingly atherogenic as CKD progresses [22, 23]. As a result, many national and international guidelines concerned with reducing this risk recommend lipid-lowering therapy for patients with pre-dialysis CKD [24, 25, 26]. Allopurinol–LDL-C reduction might increase cardiovascular risk if it spuriously lowers cardiovascular risk calculations (which rely in part on LDL-C readings) and leads to undertreatment including with statins. Alternately, allopurinol–LDL-C reduction might reduce cardiovascular risk if, like the statins, it has pleiotropic and/or direct cardiovascular benefits. Allopurinol use has been linked to a decrease in inflammatory and vascular endothelial markers, blood pressure, and rates of cardiovascular disease in numerous studies [5, 27]. Allopurinol–LDL-C reduction might also have a minimal or no net effect on cardiovascular risk if neither of the two preceding scenarios hold true, or if they exert cardiovascular effects of similar but opposing magnitudes. The many reports of neutral or conflicting results regarding allopurinol use and cardiovascular outcomes support this last scenario [28–33, 34]. Within the limits of the current study, our inability to detect differences in patient and renal survival after allopurinol use also supports a neutral impact on mortality. Finally, as most patients prescribed allopurinol do not have renal disease, efforts to examine these associations and delineate potential clinical implications in non-renal populations might also be indicated.

## LIMITATIONS

Our study has several limitations. 

First, as a post-hoc analysis of a prospective cohort study, it was not designed to assess causal associations between allopurinol use, serum urate, and lipids. Second, we did not assess potential secondary causes of dyslipidemia, such as thyroid dysfunction or alcohol consumption or diet, which could have confounded the relationship between serum urate and lipid levels. This meant we were unable to account, for example, for the strong link between adherence to specific dietary patterns and the risks for gout-related metabolic disorders, hyperuricemia, hypercholesterolemia, and metabolic syndrome [35, 36, 37]. Third, we did not have information about the allopurinol doses or patient adherence rates and are unable to comment on a possible dose response. However, by examining urate categories we were able to provide an indication of allopurinol efficacy as it relates to lipid levels. Fourth, there remains the possibility of random error in our statistical models, and some of our findings may be partially explained by regression to the mean.

## CONCLUSIONS

Allopurinol use predicted reduced total cholesterol and especially LDL-C in this large prospective cohort of patients with pre-dialysis CKD. Future studies could uncover underlying mechanisms, delineate clinical implications, and confirm these findings in this and other populations.

## Supplementary Material

sfae400_Supplemental_File

## Data Availability

The research data that support the findings of this study are not publicly available but will be shared on reasonable request to the corresponding author.
