# Supplementary material for: Allopurinol use predicts lower low-density lipoprotein cholesterol in patients with pre-dialysis chronic kidney disease—a prospective cohort study
Source: Clin Kidney J. 2024 Dec 9;18(4):sfae400. doi: 10.1093/ckj/sfae400 (PMC12209807; doi:10.1093/ckj/sfae400)
Supplement: sfae400_Supplemental_File [file sfae400_Supplemental_File.docx]

**Supplemental Table 1: Hazard Ratios and Percentage Change for Predictors of Lipid Levels and Event Outcomes (Censoring for Death or Initiation of Renal Replacement Therapy) in a Generalized Structural Equation Model (GSEM) *Among Patients Prescribed Allopurinol, Stratified by Gout Status***

|  | **Independent**  **Variable** | **Total**  **Cholesterol**  **(95% CI)**  **p-value** | **LDL-C**  **(95% CI)**  **p-value** | **HDL-C**  **(95% CI)**  **p-value** | **Triglycerides**  **(95% CI)**  **p-value** |
| --- | --- | --- | --- | --- | --- |
| % change in lipid levels | History of gout  (if present) | 3.38  (-2.97 to 3.81)  0.834 | 0.78  (-5.30 to 7.25)  0.806 | -1.04  (-5.27 to 3.38)  0.641 | 2.68  (-4.88 to 10.84)  0.498 |
| Hazard Ratios for censured events | History of gout  (if present) | 0.95  (0.46 to 1.96)  0.897 | 0.95  (0.47 to 1.07)  0.923 | 1.00  (0.49 to 2.07)  0.980 | 1.03  (0.49 to 2.16)  0.934 |

*Adjusted for age, gender, BMI, visit number (as a proxy for time), eGFR, diabetes history, serum albumin, C reactive Protein, and microalbumin/creatinine ratio, with an unstructured covariance and robust standard errors.

**Differences in lipid levels expressed as percentages and calculated using exponentials of model-predicted beta-coefficients: (e^β^ - 1) * 100). Differences in censured events expressed as Hazard Ratios and calculated by exponentiating beta coefficients (e^β^).

**Supplemental Table 2: Hazard Ratios and Percentage Change for Predictors of Lipid Levels and Event Outcomes (Censoring for Death or Initiation of Renal Replacement Therapy) in a Generalized Structural Equation Model (GSEM) *Among Patients Prescribed Allopurinol, Stratified by Gout Status and Urate Categories***

|  | **Comparison**  **groups***** | **Total Cholesterol (95% CI)**  **p-value** | **LDL-C**  **(95% CI)**  **p-value** | **HDL-C**  **(95% CI)**  **p-value** | **Triglycerides**  **(95% CI)**  **p-value** |
| --- | --- | --- | --- | --- | --- |
| % change in lipid levels | Gout, TU  versus  No Gout, TU | -0.49  (-4.18 to 3.35) 0.799 | -1.72  (-8.37 to 5.42) 0.628 | -0.27  (-4.95 to 4.64)  0.912 | 3.02  (-5.76 to 12.62)  0.513 |
|  | No Gout, HU  versus  No Gout, TU | 3.41  (1.25 to 5.61) **0.002** | 5.26  (0.45 to 10.31)  **0.032** | 2.05  (-0.30 to 4.45)  0.087 | 2.64  (-3.17 to 8.80)  0.381 |
|  | Gout, HU  versus  No Gout, TU | 4.87  (1.14 to 8.74) **0.010** | 8.97  (1.61 to -16.87)  **0.016** | 0.57  (-4.10 to 5.48)  0.813 | 5.39  (3.51 to 15.11)  0.243 |
| Hazard Ratios for censured events | Gout, TU  versus  No Gout, TU | 0.67  (0.27 to1.71)  0.410 | 0.69  (0.28 to 1.75)  0.439 | 0.73  (0.29 to 1.79)  0.485 | 0.77  (0.30 to 1.98)  0.593 |
|  | No Gout, HU  versus  No Gout, TU | 0.36  (0.98 to 1.34)  0.127 | 0.37  (0.10 to 1.36)  0.133 | 0.36  (0.10 to 1.32) 0.123 | 0.37  (0.10 to 1.38)  0.138 |
|  | Gout, HU  versus  No Gout, TU | 0.53  (0.20 to 1.37)  0.188 | 0.53  (0.21 to 1.39)  0.197 | 0.54  (0.21 to 1.38)  0.197 | 0.55  (0.21 to 1.45)  0.226 |

*Adjusted for age, gender, BMI, visit number (as a proxy for time), eGFR, diabetes history, serum albumin, c reactive protein and microalbumin/creatinine ratio, with an unstructured covariance and robust standard errors.

**Differences in lipid levels expressed as percentages and calculated using exponentials of model-predicted beta-coefficients: (e^β^ - 1) * 100). Differences in censured events expressed as Hazard Ratios and calculated by exponentiating beta coefficients (e^β^).

*** NA (no allopurinol), A (on allopurinol), TU (target urate, <6 mg/dL) and HU (high urate, ≥ 6 mg/dL)
